# Effect of short-term prehabilitation of older patients with colorectal cancer: A propensity score-matched analysis

**DOI:** 10.3389/fonc.2023.1076835

**Published:** 2023-02-16

**Authors:** Xiayun Wang, Ruizhe Chen, Lili Ge, Yifan Gu, Lin Zhang, Li Wang, Chengle Zhuang, Qian Wu

**Affiliations:** ^1^ Department of Gastrointestinal Surgery, Shanghai Tenth People’s Hospital Affiliated to Tongji University, Shanghai, China; ^2^ College of Medicine, Tongji University, Shanghai, China

**Keywords:** multimodal prehabilitation, colorectal cancer, functional capacity, enhanced recovery after surgery, older adults

## Abstract

**Objective:**

The aim of this study was to assess the impact of short-term, hospital-based, supervised multimodal prehabilitation on elderly patients with colorectal cancer.

**Methods:**

A single-center, retrospective study was conducted from October 2020 to December 2021, which included a total of 587 CRC patients who were scheduled to undergo radical resection. A propensity score-matching analysis was performed to reduce selection bias. All patients were treated within a standardized enhanced recovery pathway, and patients in the prehabilitation group received an additional supervised, short-term multimodal preoperative prehabilitation intervention. Short-term outcomes were compared between the two groups.

**Results:**

Among the participants, 62 patients were excluded; 95 participants were included in the prehabilitation group and 430 in the non-prehabilitation group. After PSM analysis, 95 pairs of well-matched patients were included in the comparative study. Participants in the prehabilitation group had better preoperative functional capacity (402.78 m vs. 390.09 m, P<0.001), preoperative anxiety status (9% vs. 28%, P<0.001), time to first ambulation[25.0(8.0) hours vs. 28.0(12.4) hours, P=0.008], time to first flatus [39.0(22.0) hours vs. 47.7(34.0) hours, P=0.006], duration of the postoperative length of hospital stay [8.0(3.0) days vs. 10.0(5.0) days, P=0.007), and quality of life in terms of psychological dimensions at 1 month postoperatively [53.0(8.0) vs. 49.0(5.0), P<0.001].

**Conclusion:**

The short-term, hospital-based, supervised multimodal prehabilitation is feasible with a high degree of compliance in older CRC patients, which improves their short-term clinical outcomes.

## 1 Introduction

Worldwide, with more than 1.9 million cases and 935.173 deaths a year, colorectal cancer (CRC) is the third most common cancer and the second leading cause of cancer death (1) for which surgery is the main treatment. However, approximately one-third of patients who undergo colorectal resection experience postoperative complications (2, 3), which delay postoperative recovery, prolong hospital stays, lead to unplanned readmissions and reduce health-related quality of life (4). More than 65% of CRC patients are older than 65 years old (5). In the elderly, there is a progressive decline in the physiological function and reserve of several organ systems (6), which affects the tolerance to surgery. Older patients have a higher risk of postoperative complications, with a consequently longer postoperative hospitalization duration and a higher 30-day mortality rate (7–9).

Higher preoperative physical capacity levels are associated with a lower risk of postoperative complications and decreases in the postoperative hospitalization duration (10). However, medical staff target the postoperative period for rehabilitation and frequently neglect the assessment of and interventions for preoperative risk factors, such as malnutrition and frailty, and mental burdens such as anxiety and depression. Patients in the postoperative phase often experience pain, weakness, lack of sleep, etc. (11), and are thus more psychologically receptive to behavioral interventions in the preoperative period when scheduled to undergo major surgery (12, 13). “Prehabilitation” refers to interventions that enable patients to withstand an incoming stressor (10), which mainly includes the assessment of the patient’s preoperative physical, nutritional, and psychological status and interventions. Kamarajah et al. (14) found that prehabilitation successfully reduces the risk of morbidity and postoperative complications. Recent studies (15, 16) have shown that multimodal prehabilitation programs, including interventions intended to enhance patients’ functional capacity (17), nutritional status (18), and psychological status (19), were more effective than a single modality. However, the current prehabilitative interventions vary in terms of duration (4-12 weeks), site (home-based or hospital-based), modality (multi- or unimodal, with different components of exercise, nutrition, psychology, etc.), intensity, and outcome indicators. Systematic reviews (20–22) have identified the significance of high-intensity, long-term, individualized prehabilitation for improving the clinical outcomes of patients. However, adherence to long-term programs remains a significant barrier with regard to prehabilitation management. Considering the imperfection of the community-hospital medical structure in China, which is characterized by an imperfect management system and is ill-equipped for prehospital patient referral, prehospital education, and prehospital optimization (23), cannot ensure the smooth implementation of prehospital prehabilitation. Furthermore, due to the fear of cancer, most patients who are diagnosed with CRC are eager to undergo surgery as soon as possible. In China, most patients with gastrointestinal cancer routinely spend 3–12 inpatient days preparing for the operation (24, 25). Therefore, it is of interest to determine whether preoperative prehabilitation within this preparatory stay would be appropriate and feasible, based on the premise of not increasing the in-hospital stay.

Thus, the aim of our study is to investigate the feasibility and effectiveness of a short-term, hospital-based, supervised multimodal prehabilitation program for older CRC patients.

## 2 Materials and methods

### 2.1 Study design and participants

This single-center, retrospective study of older CRC patients was conducted at Shanghai Tenth People’s Hospital from October 2020 to December 2021. In order to avoid intergroup contamination, patients were stratified into two groups prospectively at different periods based on whether they had implemented multimodal prehabilitation programs: the prehabilitation group included consecutively treated patients between July and December 2021, and the non-prehabilitation group of non-prehabilitation included consecutive patients who underwent surgery at the same hospital between October 2020 and June 2021, without multimodal prehabilitation programs.

Patients aged ≥65 years who were scheduled for elective CRC surgery were eligible for study participation. Patients were excluded if they (1) had a psychiatric history and could not understand instructions, (2) did not undergo surgery, (3) underwent emergency surgery, (4) had the new-adjuvant therapy before surgery; (5) were hospitalized for less than 5 days before the surgery, (6) had the American Society of Anesthesiologists (ASA) grade >III, (7) had premorbid conditions (i.e., cardiorespiratory, musculoskeletal) that contraindicated exercises, (8) received therapeutic diets or other conditions that precluded nutritional intervention, (9) had ≤50% adherence to the exercise prehabilitation program, or (10) refused to participate in this study. The study was registered at the Chinese Clinical Trial Registry (ChiCTR2000040928) and was approved by the hospital ethics committee (SHSY-IEC-4.1/20-205/01); all patients provided written informed consent for study participation.

### 2.2 Perioperative care

Both groups received the Enhanced Recovery After Surgery (ERAS) protocol in the perioperative period. Key aspects of this protocol include adequate preoperative health education, short preoperative fasting time (which included no fluid intake for 2 hours before surgery but received oral carbohydrates 2-3 hours prior to surgery), maintenance of normothermia, perioperative multimodal analgesia, removal of the catheter as early as possible, and early mobilization and feeding.

### 2.3 Prehabilitation management

In addition to standard ERAS-based care, patients in the Prehabilitation group (Prehab group) received individualized, supervised, in-hospital, and short-term multimodal prehabilitation. All patients received prehabilitation measures, including exercise, nutrition, and psychological adjustment, for at least 5 days before surgery. The program duration was adapted to the surgical schedule and the prehabilitation program was formulated and began immediately after the baseline assessment of the patient by the multidisciplinary team on the day of admission. Prior to prehabilitation, all patients received the necessary education that was provided by a multidisciplinary team, which was mainly composed of the attending physician, kinesiologist, dietician, psychologist, and nursing staff. The attending physician mainly conducted the baseline assessment of the patient, whereas the kinesiologist, dietician, and psychologist made timely program adjustments for the implementation of prehabilitation, and the nursing staff provided adequate health education and undertook data collection from the patient. Instruction booklets with details of prehabilitation and a diary were delivered to patients to record the completion of prehabilitation every day.

### 2.4 Exercise intervention

To improve physical activity, patients were required to perform aerobic, resistance, and breathing training every day. (1) Aerobic exercise consisted of a 5-min warm-up, 20-min brisk walking or cycling, and 5-min cool-down. The resting heart rate and blood pressure were recorded before all supervised sessions. The target intensity of aerobic exercise was 60–75% of the heart-rate reserve. (2) Resistance exercise mainly consisted of 25 minutes of resistance exercises using different weight sandbags, and 5 minutes of stretching. Patients were provided with four sandbags (1, 3, or 5 kg, depending on the patient’s ability) and trained in a sitting or standing position, with one sandbag in each hand, the hands held straight and parallel to the ground, and then lifted until the forearms were perpendicular to the upper arms. Two sandbags were tied to the patient’s left and right ankles, and the patients were instructed to perform straight leg raising exercises in a sitting position, with both lower limbs straight and raised as high as possible, while alternating between the two lower limbs. (3) Breathing training mainly included 10 minutes of abdominal breathing training, which was intended to strengthen the diaphragmatic muscles and improve breathing efficiency. Patients were required to inhale slowly to the maximum lung capacity through the nose, hold their breath for a short time, and then slowly exhaled all the air through the mouth. All of the abovementioned exercises were performed 3 times/per day. The patient’s first exercises were guided by a trained kinesiologist throughout the whole process, and the exercise plan was adjusted in time according to the patient’s exercise situation. During the training, all patients were supervised by the same team of trained nurses, and the procedure was stopped if the patients had any obvious discomfort, such as shortness of breath, dyspnea, or exhaustion.

### 2.5 Nutritional intervention

The dietitian provided individualized diet plans for patients to improve caloric balance, bowel movement regularity, glycemic control, etc. Correct the patient’s unhealthy eating habits by avoiding a high-calorie and, high-fat diet, eating more vegetables and fruits, and consuming more high-quality protein. A high-calorie diet was recommended for patients who did not meet their daily caloric needs. Patients with scores ≥3 on Nutritional Risk Screening 2002 (NRS 2002) (26) were identified as having a nutritional risk, and daily oral nutritional supplementation (ONS) was provided to achieve adequate protein intake (recommended intake 1.5 g/kg/d) (22). The protein supplement was ingested within 1 hour after exercise to facilitate muscle growth.

### 2.6 Psychological intervention

To alleviate negative emotions, the patients were taught how to channel their emotions and receive individualized guidance from a psychologist. Potential causes of perioperative fatigue, anxiety, and depression were discussed together by healthcare professionals. Nurses provided adequate health education to patients and corrected patients’ misconceptions. The psychologist evaluates the patient’s psychological condition, gives targeted counseling, and instructs the patient to listen to music before going to bed every day. Patients were instructed to perform 10 minutes of deep breathing exercises and meditation once a day.

### 2.7 Compliance and adherence to the program

Compliance with the multimodal prehabilitation program was deemed satisfactory when the patient preoperatively completed ≥75% of the scheduled exercise training tasks and fulfilled ≥75% of the protein supplementation intake and immunomodulatory formula. In our study, adherence to exercise training and psychological intervention was measured by calculating the percentage of actual exercise time versus program planned time, and adherence to the nutritional support was measured by the percentage of surplus and use of nutritional supplements compared to the required intake.

### 2.8 Data collection

All data were collected prospectively by two trained nurses. For each patient who was enrolled in this study, the following data were collected: demographic, perioperative, and outcome details.

Demographic data mainly included age, sex, education, occupation, marital status, body mass index (BMI), smoking, comorbidities, history of abdominal surgery, hypoalbuminemia (defined as serum albumin concentration <35 g/L), NRS-2002 score (26), and screening for frailty using the validated Modified Frailty Index (27) (score 0–1 indicates no frailty; and ≥2, indicates frailty).

Perioperative data included operative details, functional capacity, nutritional, and psychological outcomes. Operative details included tumor location, blood loss, type of surgery, surgical duration, and the ASA status. Functional parameters included grip strength, gait speed, and the 6-min walk test (6MWT) (28, 29). Grip strength was measured by using an electronic hand dynamometer (EH101; CAMRY, Guangdong Province, China), and the maximal hand strength was recorded in three consecutive tests. Gait speed was calculated by walking 6 m from the starting point at the patient’s usual speed, and an average of two measurements was taken. Furthermore, 6MWT (or 6-minute walk distance [6WMD]) was the distance that the patient walked back and forth in a 50-m corridor at the fastest walking speed in 6 minutes, and the 6MWD was the time it took for the patient to pass the 6-m distance at the fastest speed, and the results of two tests were averaged. Nutritional parameters included weight and triceps skinfold thickness. Triceps skinfold thickness was measured using a skinfold caliper, which pinches the skin and subcutaneous tissue of the patient’s right arm at the deltoid muscle (at the midpoint of the line from the crest of the shoulder to the ulnar eminence) with the fingers, hold the skin fold by placing the two jaws of the measuring instrument under the fingers, and an average of two measurements was taken. Anxiety and depression were assessed by the Hospital Anxiety and Depression Scale (a score of 0–7 indicates a negative result and ≥8 indicates anxiety/depression) (30).

Outcome data included postoperative complications within 4 weeks after surgery according to the Clavien–Dindo classification (31), the total and postoperative length of hospital stay (LOS), hospitalization costs, time to bowel function recovery, time to first ambulation, 30-day mortality, and 30-day hospital readmissions. Postoperative follow-up assessment mainly included the patients’ quality of life which was determined by using the 36-Item Short Form Survey (32) at 1, 3, and 6 months after surgery.

### 2.9 Statistical analysis

All data were collected prospectively and analyzed retrospectively. Descriptive analysis was performed on the baseline characteristics of the two groups. Categorical variables were presented as numbers (%), and numerical variables were expressed as the mean ± standard deviation (SD) or median and interquartile range (IQR) according to the distribution. To minimize intergroup bias due to the nonrandom allocation of treatments between the two groups, analyses between the prehab and non-prehab groups were performed using propensity score-matching (PSM) and multiple logistic regression. The patients’ propensity scores were calculated based on the following baseline factors: age, sex, education, occupation, marriage, BMI, smoking, NRS-2002, comorbidities, history of abdominal surgery, hypoalbuminemia, ASA, tumor location, type of surgery, blood loss, and duration of surgery. Participants in the prehab and non-prehab groups were then paired 1:1 in accordance with these propensity scores using a neighbor-matching algorithm without replacement, with a prespecified 0.02 standard deviation (33). Intergroup differences before and after the intervention in the two groups were compared using chi-square, Student’s *t*-, and nonparametric tests. Linear or logistic regression was used to compare intergroup differences in the postoperative outcomes. All data were analyzed with the SPSS Statistics version 23.0 (IBM, Armonk, NY, USA). Two-tailed *P-*values <0.05 were considered statistically significant.

## 3 Results

**Figure 1:**
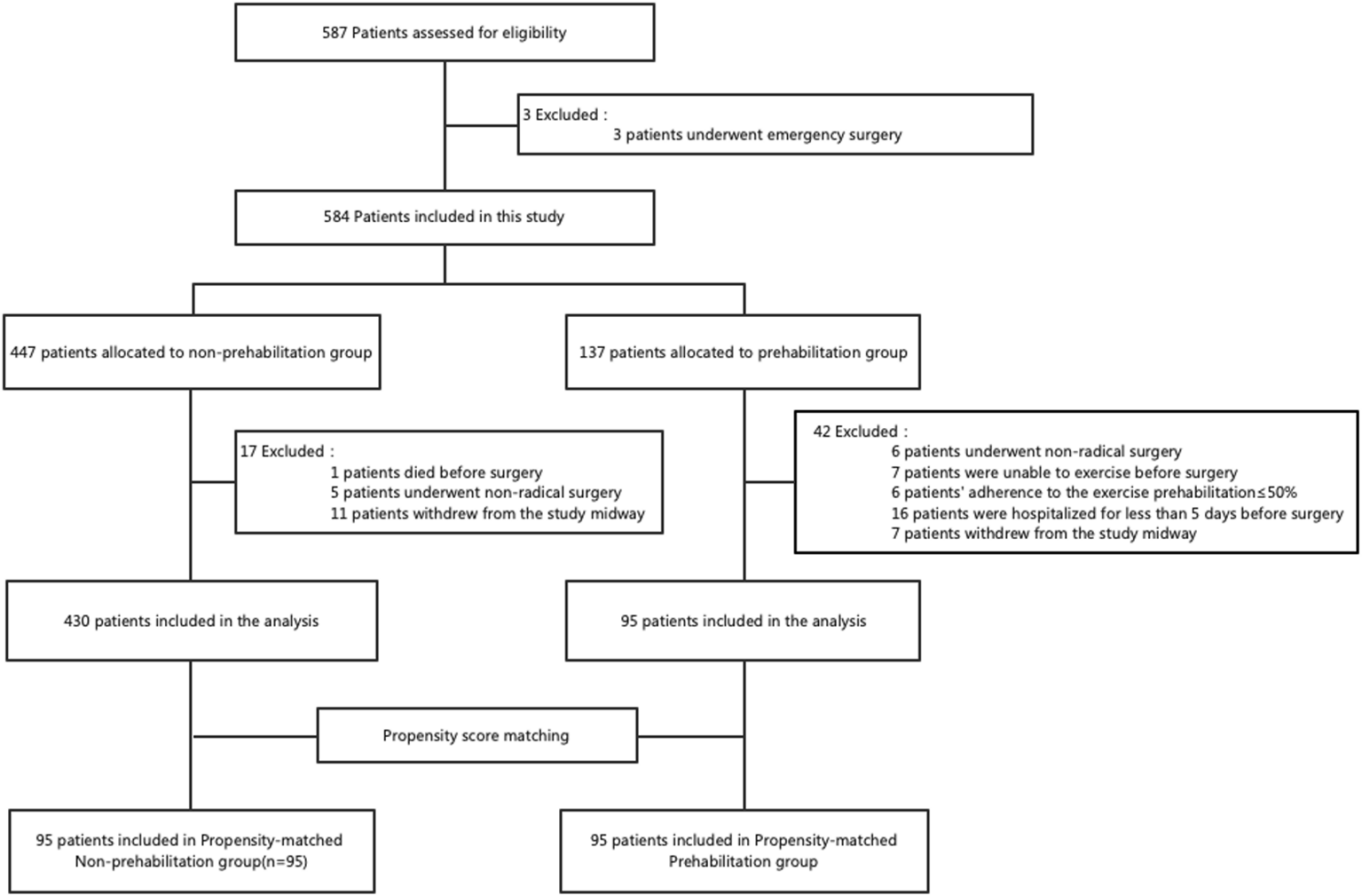
Flowchart of the patient selection process in the study.

A total of 587 CRC patients who were treated from October 2020 to December 2021 were assessed for eligibility in this study; 10 patients were excluded because they underwent emergency surgery or were unable to exercise, and the remaining 577 patients were included in this study. Subsequently, 59 patients were excluded during the study (reasons: died before surgery [n=1], underwent non-radical surgery [n=11], ≤50% adherence to the exercise prehabilitation [n=6], hospitalized for less than 5 days before surgery [n=16], and withdrew from the study midway [n=18]). Of the 18 patients who withdrew midway through the study, 5 withdrew because they perceived a lack of benefit, 2 found it difficult to adhere to the study procedures, and 11 withdrew because they refused to participate in the follow-up. Finally, 430 patients in the prehab group and 95 patients in the non-prehab group were included in the analysis. After a 1:1 ratio PSM, 95 patients were included in each of the two groups (**Figure 1**).

The participant characteristics are summarized in **Table 1**. Before matching, there were 95 patients in the prehab group and 430 patients in the non-prehab group. Patients in the prehab group were older (*P<*0.001), and had lower BMI (*P*=0.009) than participants in the non-prehab group. After the 1:1 ratio PSM, there was no significant intergroup difference (*P*>0.05) in terms of baseline and surgical characteristics.

**Table 1:** Baseline and surgical characteristics of the study cohort before and after matching.

	Unmatched comparison			Matched comparison		
	Prehab group (N=95)	Non-prehab group (N=430)	*P*-value	Prehab group (N=95)	Non-prehab group (N=95)	*P*-value
**Age, median (IQR), years**	71 (10)	67 (12)	<0.001*	71 (10)	70 (9)	0.401
Sex				0.569				0.762
Male	60 (63.2)	258 (60.0)		60 (63.2)	62 (65.3)	
Female	35 (36.8)	172 (40.0)		35 (36.8)	33 (34.7)	
Education				0.901				0.972
Primary school or less	24 (25.3)	96 (22.3)		24 (25.3)	23 (24.2)	
Junior high school	39 (41.1)	176 (40.9)		39 (41.1)	42 (44.2)	
High school	21 (22.1)	108 (25.1)		21 (22.1)	19 (20.0)	
College degree and higher	11 (11.6)	50 (11.6)		11 (11.6)	11 (11.6)	
Occupation				0.611				0.772
Retired	73 (76.8)	343 (79.8)		73 (76.8)	77 (81.1)	
Employed	7 (7.4)	31 (7.2)		7 (7.4)	6 (6.3)	
Unemployed	15 (15.8)	56 (13.0)		15 (15.8)	12 (12.6)	
Marriage				0.849				0.779
Married	89 (93.7)	409 (95.1)		89 (93.7)	91 (95.8)	
Unmarried	4 (4.2)	14 (3.3)		4 (4.2)	3 (3.2)	
Widowed	2 (2.1)	7 (1.6)		2 (2.1)	1 (1.0)	
**BMI, mean** ± **SD, kg/m^2^**	23.65 ± 0.29	24.53 ± 0.14	0.009*	23.65 ± 0.29	23.47 ± 0.34	0.839
Smoking				0.605				0.620
Yes	10 (10.5)	38 (8.8)		10 (10.5)	8 (8.4)	
No	85 (89.5)	392 (91.2)		85 (89.5)	87 (91.6)	
NRS-2002				0.867				0.881
<3	36 (37.9)	159 (37.0)		36 (37.9)	37 (38.9)	
≥3	59 (62.1)	271 (63.0)		59 (62.1)	58 (61.1)	
Comorbidities						
Hypertension (Yes)	53 (55.8)	227 (52.8)	0.596	53 (55.8)	49 (51.6)	0.561
Diabetes (Yes)	20 (20.1)	76 (17.7)	0.441	20 (20.1)	16 (16.8)	0.459
History of stroke (Yes)	12 (12.6)	46 (14.9)	0.586	12 (12.6)	11 (11.6)	0.824
Previous abdominal surgery				0.173				0.583
Yes	17 (17.9)	105 (24.4)		17 (17.9)	20 (21.1)	
No	78 (82.1)	325 (75.6)		78 (82.1)	75 (78.9)	
MFI				0.169				0.510
0–1	68 (71.6)	336 (78.1)		68 (71.6)	72 (75.8)	
≥2	27 (28.4)	94 (21.9)		27 (28.4)	23 (24.2)	
Hypoalbuminemia				0.340				0.635
Yes	30 (31.6)	115 (26.7)		30 (31.6)	27 (28.4)	
No	65 (68.4)	315 (73.3)		65 (68.4)	68 (71.6)	
Tumor location				0.148				0.162
Colon	69 (72.6)	279 (64.9)		69 (72.6)	60 (63.2)	
Rectum	26 (27.4)	151 (35.1)		26 (27.4)	35 (36.8)	
ASA status				0.403				0.410
I	27 (28.4)	98 (22.8)		27 (28.4)	21 (22.1)	
II	62 (65.3)	293 (68.1)		62 (65.3)	64 (67.4)	
III	6 (6.3)	39 (9.1)		6 (6.3)	10 (10.5)	
Blood loss, ml				0.655				0.717
<150	77 (81.1)	340 (79.1)		77 (81.1)	75 (78.9)	
≥150	18 (18.9)	90 (20.9)		18 (18.9)	20 (21.1)	
Type of surgery				0.643				0.407
Laparoscopic	91 (95.8)	416 (96.7)		91 (95.8)	93 (97.9)	
Open	4 (4.2)	14 (3.3)		4 (4.2)	2 (2.1)	
Duration of surgery, hours				0.253				0.232
<3	76 (80.0)	320 (74.7)		76 (80.0)	69 (72.6)	
≥3	19 (20.0)	110 (25.6)		19 (20.0)	26 (27.4)	

BMI, body mass index; MFI, Modified Frailty Index; NRS-2002, Nutritional Risk Screening 2002; ASA, American Society of Anesthesiologists.

Values in parentheses are percentages unless indicated otherwise.

*Statistically significant (P<0.05).

In the prehab group, the median duration of the prehabilitation program was 7 days (interquartile range [IQR], 5–12). Adherence to exercise, nutritional, and psychological prehabilitation was 83.2%, 93.7%, and 100%, respectively. Compliance with the multimodal prehabilitation program was satisfactory in 79 (83.2%) patients.

After matching, data on intergroup differences before and after intervention are presented in **Table 2**. Compared to the non-prehab group, we found that multimodal prehabilitation improved 6MWD and reduced the anxiety scores of older patients (*P*<0.05). Data on postoperative outcomes are presented in **Table 3**. The prehab group had early ambulation [25.0 (8.0) hours *vs.* 28.0 (12.4) hours, *P*=0.008], shorter first flatus time [39.0 (22.0) hours *vs.* 47.7 (34.0) hours, *P*=0.006], and postoperative LOS [8.0 (3.0) days *vs.* 10.0 (5.0) days, *P*=0.007] than the non-prehab group. With regard to the quality of life (36-Item Short Form Survey), the prehab group had higher total mental SF-36 subscale scores 1 month after surgery than the non-prehab group [53.0 (8.0) *vs.* 49.0 (5.0), *P*<0.001]. No intergroup difference was found for the other clinical outcomes.

**Table 2:** Pre- and post-intervention intergroup differences.

Variables	Prehab group (N=95)		Non-prehab group (N=95)		P_1_	P _2_	P_3_
	Time 1	Time 2	Time 1	Time 2			
**6MWD, mean ± SD, m**	389.98 ± 76.76	402.78 ± 74.71	388.92 ± 84.42	390.09 ± 81.59	0.928	0.265	<0.001^c^
**Grip strength, median (IQR), kg**	24.90 (8.40)	25.00 (10.70)	24.90 (10.80)	25.40 (11.60)	0.284	0.264	0.202
**Gait speed, median (IQR), m/s**	1.03 (0.69)	1.12 (0.65)	1.19 (0.79)	1.10 (0.74)	0.132	0.254	0.762
**weight, median (IQR), kg**	60.15 (10.10)	63.00 (10.50)	60.70 (8.30)	61.00 (9.00)	0.721	0.818	0.104
**Triceps skinfold thickness, median (IQR), mm**	13.20 (4.30)	13.20 (4.60)	13.20 (4.60)	13.20 (4.30)	0.961	0.748	0.130
**HADS-Anxiety^a^**	24 (25.3)	9 (9.5)	18 (18.9)	28 (29.5)	0.294	<0.001^c^	NA
**HADS-Depression^a^**	8 (8.4)	11 (11.6)	13 (13.7)	16 (16.8)	0.247	0.299	NA

P_1_ refers to the comparison between Time1 values in the prehab and non-prehab groups; P_2_ refers to the comparison between Time2 values in the prehab and non-prehab groups; P_3_ refers to the comparison between the difference in values between the prehab and non-prehab groups from before to after the intervention.

NA, not applicable.

**^a^** Values are expressed as number (%).

*Statistically significant (P<0.05).

**Table 3:** Intergroup differences in the postoperative short-term outcomes.

Outcomes	Prehab group (N=95)	Non-prehab group (N=95)	*P-*value
Postoperative complication^a^			0.764
None	65 (68.4)	66 (69.5)	
I	12 (12.6)	11 (11.6)	
II	12 (12.6)	15 (15.7)	
III	3 (3.2)	2 (2.1)	
IV	1 (1.1)	1 (1.1)	
V	2 (2.1)	0 (0.0)	
**Total LOS, days^b^**	16.0 (5.0)	15.0 (8.0)	0.098
**Postoperative LOS, days^b^**	8.0 (3.0)	10.0 (5.0)	0.007^*^
**Time to first ambulation, hours^b^**	25.0 (8.0)	28.0 (12.4)	0.008^*^
**Time to first flatus, hours^b^**	39.0 (22.0)	47.7 (34.0)	0.006^*^
**Time to first defecation, hours^b^**	89.0 (28.0)	89.0 (18.4)	0.104
**Hospitalization costs, yuan^b^**	70972.5 (15002.2)	66517.0 (14742.3)	0.149
**30-day mortality^a^**	2.0 (2.1)	0 (0.0)	0.155
**30-day hospital readmission^a^**	3.0 (3.2)	5.0 (5.3)	0.470
Total Physical SF-36 subscale^b^			
1-month after surgery	51.0 (5.0)	51.0 (5.0)	0.998
3-month after surgery	54.0 (9.0)	52.0 (10.0)	0.139
6-month after surgery	55.0 (16.0)	53.0 (9.0)	0.104
Total Mental SF-36 subscale^b^			
1-month after surgery	53.0 (8.0)	49.0 (5.0)	<0.001^*^
3-month after surgery	53.0 (10.0)	53.0 (9.0)	0.758
6-month after surgery	58.0 (17.0)	55.0 (15.0)	0.437

IQR, interquartile range; LOS, length of hospital days; OR, odds ratio; MD, mean difference; CI, confidence interval.

**^a^** Values are expressed as number (%).

**^b^** Values are expressed as median (IQR).

*Statistically significant (P<0.05).

## 4 Discussion

In the current study, we focused on older CRC patients and adopted an in-hospital, supervised multimodal prehabilitation to improve patient compliance and ensure effective implementation of the prehabilitation program. To reduce bias due to the differences in age, BMI, and the duration of surgery between the two groups in this non-randomized controlled study, we adjusted the unbalanced baseline characteristics between the groups by using PSM analysis to ensure the reliability of the study.

The results from this study demonstrated that a short-term, hospital-based, supervised multimodal prehabilitation significantly improved short-term surgical outcomes in older CRC patients, including preoperative functional capacity, preoperative anxiety status, time to first ambulation, time to return to bowel function, duration of postoperative LOS, and quality of life in terms of psychological dimensions at the 1-month postoperative timepoint. We suggested that the additional prehabilitation might be a beneficial factor for early recovery after colorectal surgeries in the context of the standardized enhanced recovery protocol.

Patients in the prehab group received the prehabilitation program for different durations and not all patients had satisfactory compliance with the intervention. Several previous studies, including those by Barberan-Garcia et al. (34) and Carli et al. (35), have included supervised exercise in their interventions. Adequate prehabilitation compliance is necessary to ensure the effectiveness of prehabilitation. Some of the negative findings may be related to the relatively low adherence of patients to exercise programs (36). Similar findings were found in a study of prehabilitation in frail patients (35), where the adherence to exercise was only 68%, resulting in negative results in two groups. These findings suggest that although prehabilitation can potentially improve physiological reserve and functional capacity to promote early recovery, low adherence to prehabilitation can hinder the effectiveness of prehabilitation interventions. The compliance of 83.2% in the current prehabilitation program was comparable to the compliance in the previous multimodal tele-prehabilitation program (81%) (37) and was higher than that of the community-based prehabilitation program (56%) (38). Despite a short prehabilitation day, we demonstrated some improvement in the short-term clinical outcomes of older patients with CRC, suggesting that, for patients with short preoperative duration, preoperative implementation of short-term prehabilitation is a feasible and effective option to ensure patient compliance. Considering that the duration of the prehabilitation regimen should not cause a delay in surgery and that the length of the regimen should align with cancer waiting-time targets, we demonstrated the feasibility of implementing a short-term (5–12 days) preoperative intervention, which we believe this is more clinically relevant and easy to implement in China when compared to the 4–8week duration and ancillary support that is available before elective surgery.

Pecorelli et al. (29) have shown the value of the 6MWT as a gauge of increased functional capacity. Multiple studies (22, 39) have shown the effectiveness of prehabilitation in improving preoperative 6MWT in older patients. In this study, patients in the prehab group had improved preoperative 6MWT and better postoperative ambulation, which reduced the recovery time of postoperative bowel function and postoperative LOS. Potential explanations for these findings include the possibility that short-term prehabilitation enhanced patients’ preoperative functional capacity, reduced surgical stress in patients, and faster recovery of postoperative gastrointestinal function, which led to a shorter postoperative hospital stay. Our study demonstrated that short-term prehabilitation improved preoperative physical function but not nutrition before surgery, possibly suggesting that preoperative multimodal prehabilitation does not require all programs to be conducted simultaneously. This is very informative for designing future preoperative interventions to optimize engagement throughout the preoperative period according to the waiting days before surgery. For example, nutritional prehabilitation can be started as early as possible at the time of screening or diagnosis of CRC, whereas exercise prehabilitation and psychological prehabilitation can be started later, with emphasis on supervised training for patients in short-term prehabilitation, provided that the prehabilitation outcome is met.

Our study found that the total costs during hospitalization were not higher in the prehab group than in the non-prehab group, despite the increased preoperative nutritional costs for patients in the prehab group. These economic findings suggested that this prehabilitation protocol did not increase the economic burden on the participants. Potential explanations for these findings may include the sequentially better postoperative recovery in the prehab group, which led to a shorter postoperative LOS, reduced use of medication and medical care, and consequently, conferred lower in-hospital expenses. Consistent with the findings of Frederick et al. (40) and Carli et al. (35), we did not find a decrease in postoperative complications, mortality, etc. The lack of a prehabilitation effect in these variables may be explained by the fact that the patients in the non-prehabilitation group received ERAS care rather than conventional care, and the effect of short-term multimodal prehabilitation may be limited when other aspects of perioperative care have been optimized, or given the short duration of exercise, limited effects of the selected training regimen, or various other factors.

Psychological distress is common in cancer patients. Preoperative psychological interventions appeared to improve patient-reported outcome measures in several studies (41). In addition to the surgical outcomes, we found that prehabilitation reduced preoperative anxiety and there was a progressive and significant improvement in QoL scores in the psychological dimension at the 1-month after surgery. This may indicate that patients’ active participation in the process of psychological prehabilitation may contribute to diminish the emotional distress due to their major colorectal surgery. Furthermore, anxiety is a predictor of poorer recovery and potentially decreases adherence to exercise programs (42), which also reaffirms the important role of psychological prehabilitation in multimodal prehabilitation in our study.

This study had some limitations. First, our research was performed on patients from a single center and the sample size is relatively small, the number of postoperative deaths and readmissions was low, and further confirmatory studies are required to verify these findings. Second, the two groups of patients in this study were recruited at different times and with a small sample size of patients, there were differences between the two groups at baseline characteristics, but we have used PSM analysis to balance it between the two groups. Furthermore, we considered several obstacles to recommending prehabilitation for a high-risk population in terms of the need for pre-exercise evaluations and the risk for low adherence, and accordingly excluded some patients with a higher postoperative risk; therefore, our findings may not be generalizable to these high-risk populations.

In conclusion, the findings of our study demonstrated that meaningful changes in capacity function and clinical outcomes can be achieved with short-term, hospital-based, supervised multimodal prehabilitation in older patients who were scheduled to undergo radical resection of CRC. Furthermore, we suggested the importance of supervising patients during the prehabilitation process to improve the clinical outcome.

## Data availability statement

The raw data supporting the conclusions of this article will be made available by the authors, without undue reservation.

## Ethics statement

The studies involving human participants were reviewed and approved by The Ethics Committee of Shanghai Tenth People’s Hospital. The patients/participants provided their written informed consent to participate in this study.

## Author contributions

Study design: QW and CZ. Acquisition of data: XW, LZ, YG, and LW. Analysis of data: XW, RC, and LG. Manuscript preparation: XW. All authors contributed to the article and approved the submitted version.
